# Impact of Prenatal Exposure to Maternal Diabetes and High-Fat Diet on Postnatal Myocardial Ketone Body Metabolism in Rats

**DOI:** 10.3390/ijms24043684

**Published:** 2023-02-12

**Authors:** Prathapan Ayyappan, Tricia D. Larsen, Tyler C. T. Gandy, Eli J. Louwagie, Michelle L. Baack

**Affiliations:** 1 Environmental Influences on Health and Disease Group, Sanford Research, Sioux Falls, SD 57104, USA; 2 Sanford School of Medicine, University of South Dakota, Sioux Falls, SD 57105, USA

**Keywords:** cardiovascular disease, diabetic cardiomyopathy, ketone bodies, maternal diabetes, high-fat diet, myocardial ketone metabolism, extracellular flux analyses

## Abstract

Infants exposed to diabetic pregnancy are at higher risk of cardiomyopathy at birth and early onset cardiovascular disease (CVD) as adults. Using a rat model, we showed how fetal exposure to maternal diabetes causes cardiac disease through fuel-mediated mitochondrial dysfunction, and that a maternal high-fat diet (HFD) exaggerates the risk. Diabetic pregnancy increases circulating maternal ketones which can have a cardioprotective effect, but whether diabetes-mediated complex I dysfunction impairs myocardial metabolism of ketones postnatally remains unknown. The objective of this study was to determine whether neonatal rat cardiomyocytes (NRCM) from diabetes- and HFD-exposed offspring oxidize ketones as an alternative fuel source. To test our hypothesis, we developed a novel ketone stress test (KST) using extracellular flux analyses to compare real-time ß-hydroxybutyrate (βHOB) metabolism in NRCM. We also compared myocardial expression of genes responsible for ketone and lipid metabolism. NRCM had a dose-dependent increase in respiration with increasing concentrations of βHOB, demonstrating that both control and combination exposed NRCM can metabolize ketones postnatally. Ketone treatment also enhanced the glycolytic capacity of combination exposed NRCM with a dose-dependent increase in the glucose-mediated proton efflux rate (PER) from CO2 (aerobic glycolysis) alongside a decreased reliance on PER from lactate (anaerobic glycolysis). Expression of genes responsible for ketone body metabolism was higher in combination exposed males. Findings demonstrate that myocardial ketone body metabolism is preserved and improves fuel flexibility in NRCM from diabetes- and HFD-exposed offspring, which suggests that ketones might serve a protective role in neonatal cardiomyopathy due to maternal diabetes.

## 1. Introduction

Diabetic pregnancy, especially along with a maternal high-fat diet (HFD), exposes the developing fetus to excess circulating metabolic fuels including glucose, fatty acids and ketones, which can ultimately alter metabolism and growth during critical windows of development, resulting in both short- and long-term consequences for infants [1,2,3]. Specifically, infants exposed to maternal diabetes or maternal obesity are at higher risk of heart disease at birth and early morbidity from cardiovascular disease (CVD) as adults [4,5,6,7,8]. We created a rat model to understand mechanisms of pathogenesis and showed that late-gestation diabetes, especially alongside a maternal HFD, incites mitochondrial dysfunction, impaired cardiomyocyte bioenergetics, cardiac hypertrophy, and diastolic and systolic dysfunction in newborn offspring [9,10]. Specifically, we found that maternal diabetes and HFD alters mitochondrial number, dynamics, and ultrastructure to cause these cardiometabolic consequences [10,11,12]. Importantly, maternal HFD exacerbated cardiomyopathy and perinatal mortality in offspring born to diabetic mothers (ODM) through exaggerated maternal hyperlipidemia and offspring hyperinsulinemia, myocardial lipid droplet accumulation, and oxidative stress [10,13]. Like in humans, cardiac dysfunction in our model initially improves after birth, but reappears with changing metabolic demands in an age- and sex-specific manner, likely due to programmed complex I dysfunction followed by exaggerated mitochondrial biogenesis, oxidative stress, and impaired cell survival following metabolic stress [13,14]. Given our findings alongside emerging evidence regarding the importance of ketone body metabolism in the adult failing heart [15,16], it is important to know whether developing cardiomyocytes exposed to fetal overnutrition have impaired ketone metabolism. 

Ketone bodies, including acetoacetate, acetone, and β-hydroxybutyrate (βHOB), are produced when energetic demands cannot be met by the metabolism of more readily available glucose and fatty acids. Ketone production increases moderately during physiologic conditions such as fasting, prolonged exercise, ketogenic diet, and pregnancy. Normally, circulating levels of ketones including βHOB, which accounts for 70% of the total ketones, are <0.5 mM, but hyperketonemia occurs when levels are >1.0 mM and ketoacidosis occurs when levels are over >3.0 mM [15]. Pregnancy increases ketone body production, and this is exaggerated in diabetic pregnancy [15]. Although oxidation of ketone bodies plays a significant role in energy metabolism during multiple physiological states including the neonatal period [17], in utero exposure to excess circulating fuels during diabetic pregnancy incites myocardial insulin resistance, which limits fuel flexibility and could impair ketone utilization in ODM as well.

Alterations in myocardial substrate utilization and energy metabolism is a well-known contributor to the pathogenesis of adult heart disease, especially in subjects with diabetes and obesity [18,19]. Impaired insulin signaling and increased lipolysis in diabetes leads to increased myocardial fatty acid oxidation (FAO), oxidative stress, and eventually left ventricular dysfunction. This can happen outside of other vascular risks, which is a hallmark of diabetic cardiomyopathy [20]. Under pathological conditions, the failing heart relies on ketone bodies as a source of energy [16,21]. However, Brahma, et al. recently demonstrated that increased glucose availability in diabetes attenuates myocardial ketone body utilization by suppressing cardiac ketolytic utility [22]. Our previous studies demonstrate that late-gestation diabetes and HFD not only exposes the developing fetus to maternal hyperglycemia and hyperlipidemia, but also to hyperketonemia; exposed newborns exhibit myocardial insulin resistance that is similar to what is found in adult diabetic cardiomyopathy, with a metabolic switch from glycolysis to gluconeogenesis [23] and impaired glycolytic and fatty acid oxidation capacity that is in part due to complex I dysfunction [9,13]. Importantly, complex I is also critical for ketone body metabolism. Given these findings, it is important to understand the effects of prenatal exposure to diabetes and hyperlipidemia on myocardial ketone body metabolism. 

The objective of this study was to determine whether neonatal rat cardiomyocytes (NRCM) exposed to the combination of maternal diabetes and HFD in utero can oxidize ketones as an alternative fuel source. To test this, we developed a ketone stress test (KST) using extracellular flux analyses that measures real-time βHOB metabolism in primary isolated NRCM. We also measured the expression of various genes responsible for ketone body metabolism and FAO in normal and exposed neonatal hearts.

## 2. Results

### 2.1. Ketone Stress Test (KST) in Primary Isolated Cardiomyocytes 

#### 2.1.1. Maximal and Spare Respiratory Capacity Increase Directly with Increasing Concentrations of Ketone

**Figure 1:**
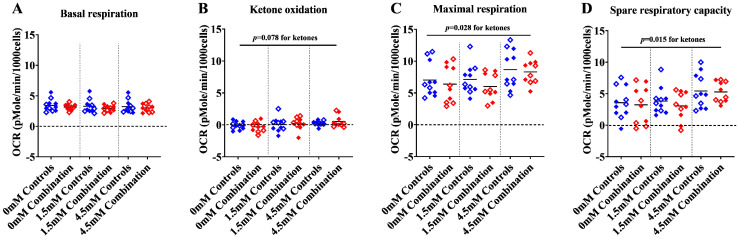
Respiratory capacity increased with increasing concentration of βHOB in both control and combination exposed cardiomyocytes. (**A**) Basal respiration, (**B**) Ketone oxidation, (**C**) Maximal respiration, and (**D**) Spare respiratory capacity in control and combination exposed NRCM. Bars represent mean OCR (n = 10–11 litter per group). Blue color indicates controls and red color indicates combination exposed. Open symbols in the graph indicate males and filled symbols indicate females. Ketone effect is noted with *p* values, as shown in the figures; there was no group or interaction effect, as determined by two-way ANOVA. mM refers to the progressive concentrations of βHOB supplied.

Respiratory capacities were measured in the presence of increasing concentrations of βHOB to determine whether control and diabetes + HFD (combination) exposed NRCM increase respiration in a dose-dependent manner. Neither basal respiration (Figure 1A) nor non-mitochondrial respiration (Figure S1A) in quiescent NRCM were significantly different between groups. In the presence of UK5099, a pyruvate inhibitor used to encourage ketone flux, NRCM were given 0 mM, 1.5 mM, or 4.5 mM βHOB then ATP production was uncoupled with FCCP to drive maximal respiration. The effects of UK5099 alone and with βHOB are detailed in Supplementary Table S5. Overall, in quiescent NRCM, OCR did not change when UK5099 was used to inhibit the mitochondrial pyruvate carrier. Ketone oxidation, defined as the ability of quiescent NRCM to utilize ketones without respiratory stimulation, was estimated as the net change in OCR from baseline to the addition of pyruvate inhibitor (UK5099) and βHOB before the addition of FCCP. While there was very little increase in OCR from the baseline, both control and combination exposed NRCM trended towards a dose-dependent increase in OCR with an increasing concentration of ketones, as shown in Figure 1B. Maximal respiration was measured after stimulating NRCM with FCCP, and spare respiratory capacity was measured as the difference between maximal and basal OCR. Control and combination exposed NRCM both had a significant dose-dependent increase in maximal respiration and spare respiratory capacity with increasing concentrations of βHOB (Figure 1C,D). Findings demonstrate that ketones can be a fuel source for NRCM, even those exposed to maternal diabetes + HFD. 

#### 2.1.2. The Presence of Ketones Improves Glycolysis in Diabetes + HFD-Exposed NRCM 

**Figure 2:**
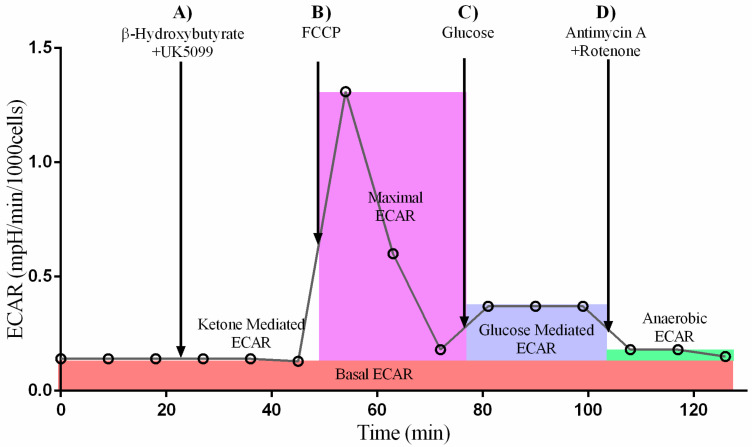
Schematic representation of the ketone stress test (KST) for assessing glycolysis in terms of extracellular acidification rate (ECAR).

**Figure 3:**
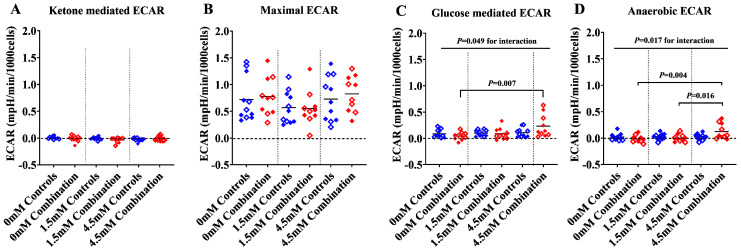
Ketones improve glucose mediated and anaerobic ECAR in combination exposed, but not in control, NRCM. (**A**) Ketone mediated ECAR, (**B**) Maximal ECAR, (**C**) Glucose mediated ECAR, and (**D**) Anaerobic ECAR in control and combination exposed NRCM. Bars represent mean ECAR (n = 10–11 litter per group). Blue color indicates controls and red color indicates combination exposed. Open symbols in the graph indicate males and filled symbols indicate females. Interaction effect was determined by two-way ANOVA, and the group differences remain significant only in combination exposed NRCM by one-way ANOVA with *p* values, as shown in the figures. mM refers to the progressive concentration of βHOB supplied.

To find out whether ketones competitively inhibit or promote glycolytic rates, we also analyzed ECAR in quiescent NRCM at the baseline, after UK5099 with or without βHOB (ketone-mediated ECAR), FCCP (maximal ECAR), glucose (glucose-mediated ECAR), and antimycin + rotenone (anaerobic ECAR). Although ECAR is considered the best estimate for glycolysis, the data should be interpreted with the knowledge that extracellular acidification depends on contributions from both lactate (anaerobic) and CO_2_ (aerobic). In this study, combination exposed NRCM tended to have a higher baseline ECAR than controls (*p* = 0.136 by *t*-test). The effects of UK5099 alone and with βHOB are detailed in Supplementary Table S6. Although UK5099, which blocks the mitochondrial pyruvate carrier, is expected to shift bioenergetics towards anaerobic glycolysis, ECAR in NRCM was not significantly different following UK5099, regardless of ketone administration. As demonstrated in a representative output graph in Figure 2, ECAR peaked after injection of FCCP, a respiratory uncoupler. This finding substantiates previous experiments, which showed that FCCP stimulates maximal ECAR in primary cardiomyocytes. This is in contrast to other cell types, which typically reach maximal ECAR in response to oligomycin, an ATP synthase inhibitor [9,13,14]. We also injected glucose after FCCP to assure that the substrate was present for glycolysis; we considered this response to be glucose mediated ECAR. Although we found no significant differences in ketone-mediated or maximal ECAR, exposure to ketones significantly increased both glucose-mediated ECAR and anaerobic ECAR in combination exposed NRCM, but not in healthy controls (Figure 3A–D). Overall, diabetes + HFD-exposed males had the greatest ability to respond to ketones, as detailed below (Supplementary Tables S7 and S8).

#### 2.1.3. Ketones Improve Proton Efflux Rate (PER) in Diabetes + HFD-Exposed NRCM

**Figure 4:**
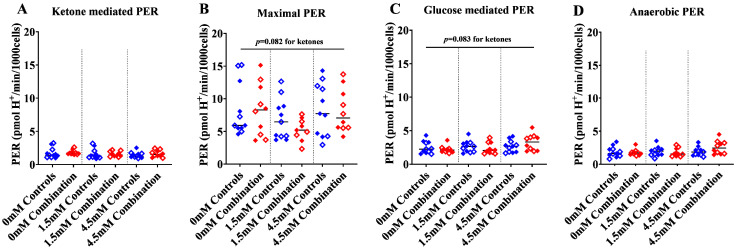
Maximal and glucose mediated total proton efflux rate (PER) tended to increase with increasing concentrations of βHOB. (**A**) Ketone mediated PER_total_, (**B**) Maximal PER_total_, (**C**) Glucose mediated PER_total_, and (**D**) Anaerobic PER_total_ in control and combination exposed NRCM. Bars represent mean PER (n = 10–11 litter per group). Blue color indicates controls and red color indicates combination exposed. Open symbols in the graph indicate males and filled symbols indicate females. Ketone mediated effect was determined by two-way ANOVA with *p* values as shown. mM refers to the progressive concentration of βHOB supplied.

**Figure 5:**
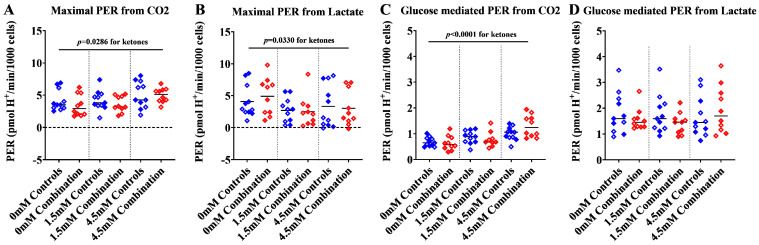
Maximal and glucose mediated proton efflux rate from CO_2_ (PER_CO2_) increased with increasing concentrations of βHOB in both control and combination exposed cardiomyocytes. (**A**) Maximal PER_CO2_, (**B**) Maximal PER from lactate (PER_Lactate_), (**C**) Glucose mediated PER_CO2_, and (**D**) Glucose mediated PER_Lactate_ in control and combination exposed NRCM. Bars represent mean PER (n = 10–11 litter per group). Blue color indicates controls and red color indicates combination exposed. Open symbols in the graph indicate males and filled symbols indicate females. Ketone effect was determined by two-way ANOVA with *p* values above straight bars; group differences as determined by one-way ANOVA are shown with *p* values above footed bars. Significance accepted at *p* < 0.05. mM refers to the progressive concentration of βHOB supplied.

**Figure 6:**
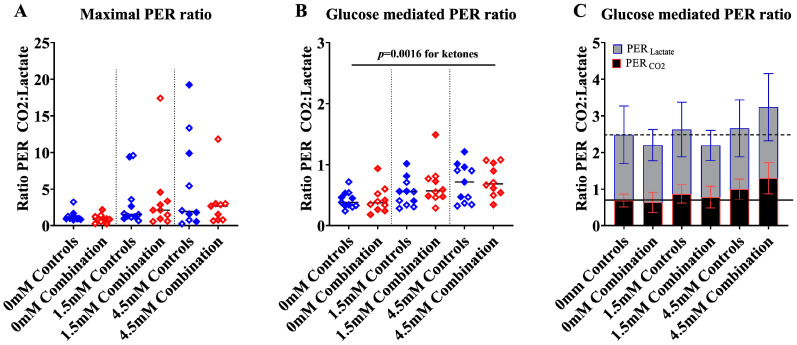
The ratio of glucose mediated PER_CO2_ and PER_Lactate_, a marker of aerobic glycolysis, increased with increasing concentration of βHOB in both control and combination exposed cardiomyocytes. (**A**) Maximal and (**B**) glucose-mediated PER_CO2_:PER_Lactate_ ratio for control and combination exposed NRCM. Bars represent mean ratio (n = 10–11 litter per group). Blue color indicate controls and red color indicate combination exposed. Open symbols in the graph indicate males and filled symbols indicate females. Ketone effect was determined by two-way ANOVA and the significance accepted at *p* < 0.05. (**C**) Bar graphs illustrate the combined contributions of PER_CO2_ (black with red border) and PER_Lactate_ (grey with blue border) to the PER_Total_ in control and combination exposed NRCM. Bars represent the mean ± SD. For illustration purposes, the solid line highlights the mean PER_CO2_ and the dashed line highlights the mean PER_Lactate_ for control NRCM that were not administered βHOB. mM refers to the progressive concentration of βHOB supplied.

To further assess the contribution of lactate (anaerobic) and CO_2_ (aerobic) flux to ECAR, we analyzed proton efflux rates (PER), which is the number of protons exported from cells over time. PER serves as a valuable tool for understanding glycolysis and fuel flexibility under various conditions. Total PER at baseline (Figure S1B), after βHOB, FCCP, and rotenone/antimycin A were not statistically different between groups; however, ketones tended to increase the maximal and glucose-mediated PER (*p* = 0.08 by two-way ANOVA) especially in combination exposed NRCM given 4.5 mM βHOB (Figure 4A–D). Interestingly, when the total PER was delineated by contribution, there was a dose-dependent increase in maximal PER from CO_2_ (aerobic respiration) alongside a dose-dependent decline in maximal PER from lactate (anaerobic glycolysis) with increasing concentrations of βHOB, and this increase was most robust in combination exposed NRCM (Figure 5A,B). Interestingly, when glucose was given as a substrate, combination exposed NRCM treated with ketones had a dose-dependent increase in glucose-mediated PER from CO_2_ (aerobic respiration), but no corresponding decline in PER from lactate (anaerobic glycolysis), which suggests that ketones enhance the ability to metabolize glucose (Figure 5C,D). Indeed, combination exposed NRCM treated with 4.5 mM had significantly higher glucose-mediated PER from CO_2_ than controls (*p* = 0.0002) and combination exposed NRCM that were not given ketones (*p* < 0.0001 by one-way ANOVA). The net effect of ketones on aerobic and anaerobic capacity in control and combination exposed NRCM is further illustrated in Figure 6A–C, where graphs show a rising ratio of glucose-mediated PER from CO_2_/lactate in the presence of ketones. Overall, our KST shows that combination exposed NRCM have improved maximal and glucose-mediated aerobic flux in the presence of ketones.

#### 2.1.4. Ketones may Enhance Fuel Flexibility More Robustly in Male NRCM

Although the KST was not originally designed to examine sex-specific differences, sex is a known biological variable in developmental origins of health and disease (DOHaD) and so post-hoc analyses were done. Sex-specific comparisons of NRCM bioenergetics are detailed in Supplementary Tables S7 and S8. When analyzing males and females separately, there were no significant group differences in respiration. However, combination exposed male NRCM treated with 4.5 mM of βHOB had a two-fold higher glucose-mediated OCR compared to those not given βHOB, which trended higher even at low numbers (*p* = 0.088, n = 5–6/group). This ketone-mediated increase in respiration is likely the result of both ketone oxidation and improved aerobic glycolysis because high dose ketones also led to a more than two-fold increase in glucose-mediated ECAR, which did reach statistical significance (*p* = 0.049) by one-way ANOVA (Supplementary Table S7). Combination exposed female NRCM also tended to increase glucose-mediated OCR with 4.5 mM βHOB treatment, but less robustly. Anaerobic ECAR also increased significantly with 4.5 mM of βHOB in combination exposed female NRCMs (Supplementary Table S7). When comparing male vs. female KST results, normal male NRCM had lower anaerobic ECAR compared to females (Table S8), but maternal diabetes + HFD increased anaerobic glycolysis in males, but lowered it in females; therefore, there were no sex-related differences in the combination exposed group. Overall, findings suggest that combination exposed males may have a greater bioenergetic response to ketones as detailed in Supplementary Tables S7 and S8.

### 2.2. Maternal Diabetes + HFD Dysregulates Genes Involved in Myocardial Ketone Body Metabolism in a Sex-Divergent Manner

**Figure 7:**
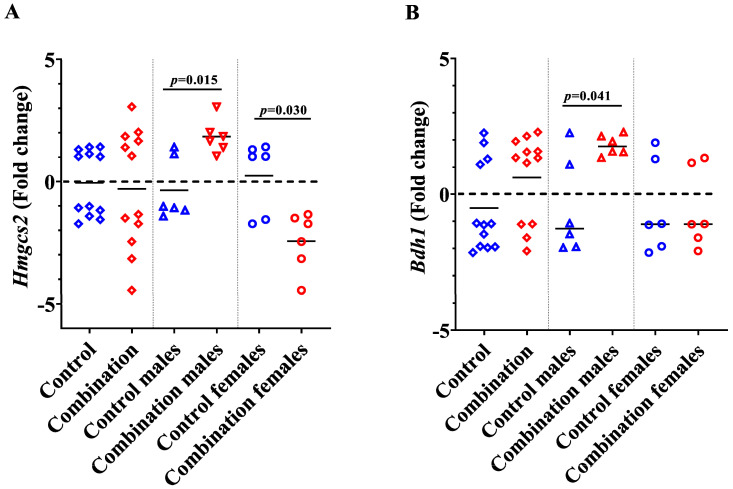
Expression of genes involved in ketone body metabolism increase in combination exposed males. (**A**) Expression of *Hmgcs2* and (**B**) *Bdh*1 in the neonatal heart of control and combination groups. Bars represents mean fold change (n = 12). Blue color indicate controls and red color indicate combination exposed. Bars represent mean fold change and the significance accepted at *p* < 0.05.

To determine the effects of maternal diabetes + HFD on levels of enzymes needed for ketone body metabolism, we examined the gene expression of 3-hydroxy-3-methylglutaryl-CoA synthase 2 (*Hmgcs2*) and β-hydroxy butyrate dehydrogenase (*Bdh1*) in neonatal myocardium (Figure 7A,B). HMGCS2, the mitochondrial protein encoded by the gene *Hmgcs2*, is involved in the synthesis of ketone bodies [22]. Interestingly, we found sex-divergent differences in *Hmgcs2* expression following maternal diabetes + HFD exposure. Compared to their control counter parts, combination exposed females had significantly lower myocardial expression of *Hmgcs2* (*p* = 0.030), while males had significantly higher expression (*p* = 0.015). BDH1, encoded by the gene *Bdh1*, is an important enzyme responsible for the catabolism of βHOB into acetyl-CoA [22]. Again, stratification by sex revealed significantly higher *Bdh1* expression in the male offspring exposed to maternal diabetes + HFD compared to their healthy male counterparts (*p* = 0.041), while *Bdh1* expression in the female offspring was not different between groups (>0.999). Overall, sex-related differences in *Hmgcs2* and *Bdh1* expression are found only in diabetes + HFD-exposed myocardium, but not in controls (Table S9). Expression differences may explain why NRCM from combination exposed males had the greatest bioenergetic response to ketones.

### 2.3. Maternal Diabetes + HFD Increases Pparg and Pgc1a Expression in Newborn Offspring Hearts

**Figure 8:**
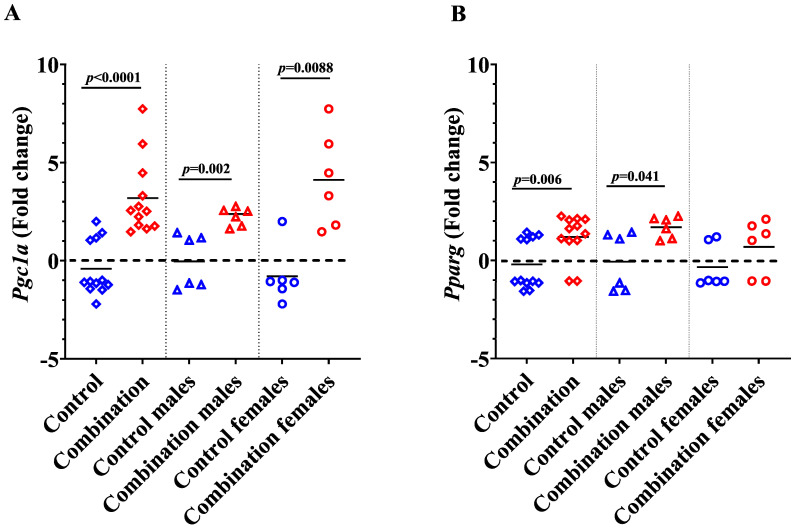
Maternal diabetes and HFD augment the myocardial expression of *Pgc1a* and *Pparg*. (**A**) Expression of *Pgc1a*, and (**B**) *Pparg* in the neonatal heart of control and combination groups. Bars represent mean fold change (n = 12). Blue color indicates controls and red color indicates combination exposed. Bars represent mean fold change and the significance accepted at *p* < 0.05.

Peroxisome proliferator-activated receptor γ (PPARG) and its coactivator-1 (PGC-1a) are transcriptional regulators of multiple genes that mediate mitochondrial biogenesis, fatty acid (FA) transport, FA utilization, and oxidative stress [24,25]. We found that the *Pgc1a* expression was significantly higher in combination exposed neonatal hearts (*p* ≤ 0.0001) compared to healthy controls (Figure 8A). Stratification by sex demonstrated an increased expression of *Pgc1a* in both the combination exposed male (*p* = 0.002) and female (*p* = 0.008) offspring.

Peroxisome proliferator-activator receptors (PPARs) are nuclear receptors that are subject to transcriptional coactivation by *Pgc1a* to play a crucial role in energy homeostasis and metabolism [26]. Among different subtypes, *Pparg* in the heart is activated by HFD, which reportedly causes lipotoxicity and myocardial dysfunction, but also activates ketogenic enzymes [27]. We found that the hearts of neonates exposed to maternal diabetes + HFD had significantly higher expression of *Pparg* compared to healthy controls (*p* = 0.006) (Figure 8B). Stratifying by sex revealed that combination exposed male hearts had a more robust increase (*p* = 0.041) in *Pparg* than females (*p* = 0.121) compared to their respective controls.

### 2.4. Diabetes + HFD Increases Expression of Cpt1a in the Neonatal Heart

**Figure 9:**
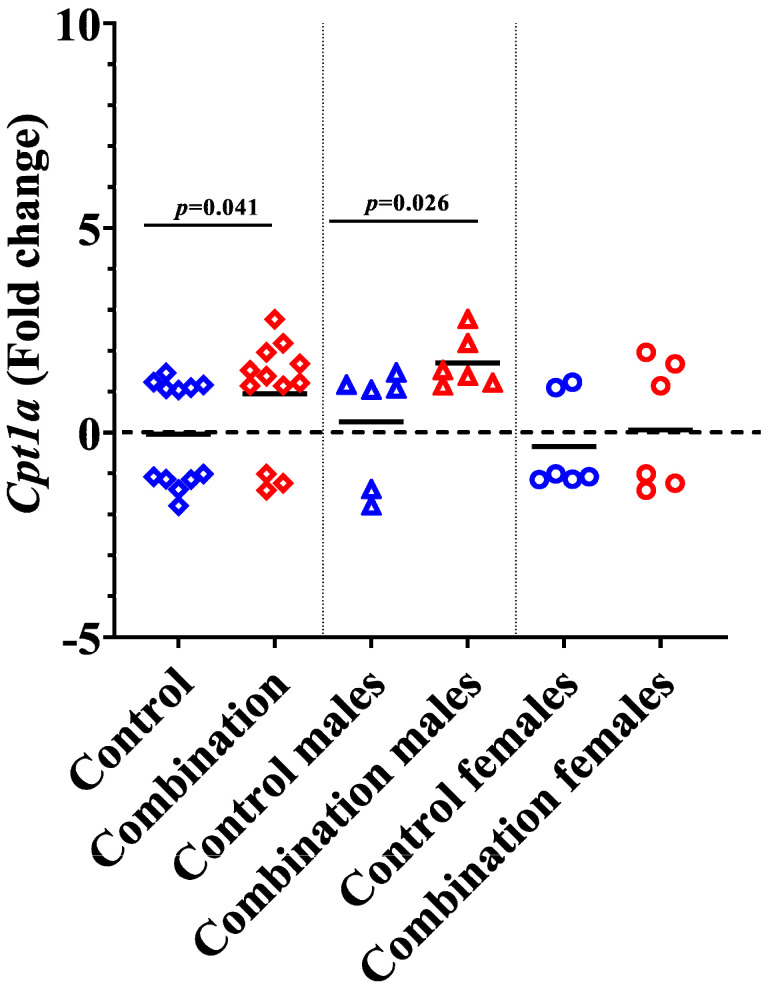
Combination exposed males have increased myocardial expression of *Cpt1a*. Bars represents mean fold change (n = 12). Blue color indicates controls and red color indicates combination exposed. Bars represent mean fold change and the significance accepted at *p* < 0.05.

The enzyme carnitine palmitoyl transferase 1 (CPT1) facilitates the transport of long-chain fatty acids from the cytoplasm to the mitochondria for β-oxidation [28]. *Cpt1a* is the predominant isoform of CPT1 in the heart at birth [29]. In our study, myocardial *Cpt1a* expression was higher in offspring exposed to maternal diabetes + HFD (*p* = 0.041) compared to controls (Figure 9). Interestingly, this was primarily due to higher expression in males (*p* = 0.026), whereas females did not have significant differences related to in utero exposure (*p* = 0.731).

## 3. Discussion

The heart is well-known for its metabolic flexibility. Indeed, the ability to switch the utilization of one substrate over the other under physiological conditions is key to meeting the high energetic demands for continuous contractile function, despite variable states of supply and demand [30,31]. The fetal heart tends towards anaerobic glycolytic metabolism due to the in utero physiology with relative hypoxia, lower cardiac demand, and continuous fuel supply from maternal circulation [32]. At birth, the neonate is exposed to an oxidative burst as it takes its first breaths. Afterload and cardiac output increase dramatically and the continuous fuel supply is interrupted by clamping the umbilical cord. These normal physiological changes incite a shift in myocardial metabolism towards oxidative phosphorylation, which requires postnatal mitochondrial biogenesis and reticulum networking [33]. Pathological conditions can disrupt this normal transition at birth. It is well known that fetal exposure to maternal diabetes increases the risk of ventricular hypertrophy and cardiomyopathy at birth, followed by a period of improvement, then a risk of early CVD as an adult [4,6]. Our rat model exposes the developing fetus to excess circulating fuels (glucose, fatty acids, and ketones), spurring fetal hyperinsulinemia, impaired myocardial metabolism, lipid accumulation, diastolic and systolic dysfunction, and increasing perinatal mortality through lipotoxic and mitochondria-mediated mechanisms [9,12,13,23]. Specifically, glucolipotoxicity impairs oxidation of complex I fuels in NRCMs followed by exaggerated mitochondrial biogenesis, oxidative stress, and faster cell death to cause biphasic cardiac disease, just like in humans [13]. 

The heart requires fuel flexibility to maintain contractile function, so it metabolizes many substrates. The heart is considered one of the highest ketone-utilizing tissues. Myocardial ketone metabolism is especially critical when there is decreased availability of other substrates [17], as found in the diabetes-exposed and insulin-resistant heart, which has impaired glucose uptake and reduced metabolic flexibility [34]. Ketones produce ATP more efficiently than glucose or fatty acid and recent studies suggest that the failing heart benefits from ketone bodies as an energy source [16,21]. βHOB, acetoacetate, and acetone are the three primary ketones in circulation, whereas βHOB is found in the highest levels [15,35]. Ketone bodies are products of fatty acid oxidation. They are synthesized via ketogenesis, then utilized within the tissues via the ketolysis pathway. Utilization of ketones under normal physiological conditions is low. However, in adult diabetic cardiomyopathy, utilization of ketones improves the prognosis of heart failure while impaired ketone utilization worsens the prognosis [31]. Given the fact that maternal diabetes + HFD cause neonatal cardiomyopathy that is similar to adult diabetic cardiomyopathy, it is important to know whether complex I dysfunction found in the diabetes-exposed neonatal heart impairs ketone body metabolism or whether ketones, which are higher in circulation during diabetic pregnancy, can be utilized by the offspring’s developing heart, especially postnatally when the continuous maternal–fetal glucose supply is interrupted by birth.

The present study used a novel KST or modified extracellular flux analyses to measure real-time βHOB metabolism in NRCM exposed to maternal diabetes and HFD. To our knowledge, this is the first study that reports the ability of the neonatal heart exposed to maternal diabetes and HFD to metabolize ketone bodies. The key finding of the study is that ketones increase respiratory capacity in a dose-dependent manner, both in controls and combination exposed NRCM. This demonstrates that ketones can serve as an alternative fuel source for the neonatal heart, even after diabetes exposure. We also showed that combination exposed NRCM, but not controls, had a ketone-dependent increase in glucose-mediated PER from CO_2_ (aerobic glycolysis), and there was less reliance on anaerobic glycolysis during metabolic stress with FCCP. This suggests that despite previously reported complex I dysfunction [13], NRCM exposed to maternal diabetes and HFD have an enhanced ability oxidize ketones compared to controls.

We also found that the expression of genes responsible for ketone body metabolism increase robustly in male offspring. This is of particular interest because males are notoriously at greater risk for cardiac consequences following in utero exposure to maternal diabetes + HFD [10,12,13,23]. While our current study was not specifically powered to determine differences in each sex, we feel that it is important to show the trends. Sex differences could be due to multiple factors such as hormonal influences, sex-regulated placental fuel transport, or epigenetic and mitochondrial variability [36,37,38,39,40]. Our previous studies consistently show a sex-specific difference in the exposure-related mitochondrial response, with females being relatively cardioprotected through better mitochondrial quality control while males had faster mitochondria-mediated cell death under metabolic stress [10,12,13]. The balance between mitophagy and mitochondrial biogenesis is influenced by *Pparg* and its coactivator *Pgc1a*, which were also upregulated in combination exposed neonatal hearts. *Pparg* is activated by free fatty acids, which trigger the increased translation of proteins needed for fatty acid uptake, formation of triglycerides, and their storage in lipid droplets [41]. While this may be a normal physiologic response in myocardium, excess fuel exposure over an extended period of time can lead to detrimental effects. Others have reported that HFD induces myocardial *Pparg* and fatty acid oxidation, leading to an increased reliance on FA metabolism, ketogenesis, reduced myocardial efficiency, and increased oxygen consumption resulting in lipotoxicity-mediated heart failure [27,42,43]. As a coactivator of *Pparg*, *Pgc1a* is involved in many overlapping cellular pathways, especially in mitochondrial biogenesis [44], which can exacerbate oxidative stress and lipotoxicity.

Sex steroid hormones could be one of the reasons for the observed sex-specific differences in *Pparg* mRNA upregulation in combination exposed offspring. It was previously shown that the administration of estradiol to ovariectomized mice exhibits a reduced level of *Pparg* mRNA expression in the adipose tissue [45]. Estrogen receptors can inhibit ligand-induced activation of *Pparg*, and the induction of estrogen receptor β (*Erb*) is stronger in the hearts of females than in males [46,47]. It is interesting to note that the *Erb*-selective ligands have a *Pgc1a*-dependant inhibitory effect on *Pparg* activity [46]. Our results are in line with these reports and also with another study where *Pparg* activation was directly associated -with upregulation of *Hmgcs2* in male animals [27]. While increased expression of *Hmgcs2* and *Bdh1* is likely a compensatory and adaptive mechanism, this interesting, sex-specific effect may offer hope for the therapeutic response to ketones in males that are more likely affected. Here, we also show a significant increase of myocardial *Cpt1a* expression in combination exposed offspring, which may further exaggerate lipotoxicity. Others demonstrated that diabetes can increase the gene expression of *Cpt1a* in the heart [48]. Expression of *Cpt1a* in the heart is also stimulated by *Pgc1a* [49]. Interestingly, all of these genes play an important role in myocardial development and metabolic flexibility that is important to life-long cardiac health.

While our findings suggest that ketones may be beneficial for insulin-resistant neonatal hearts, others have reported detrimental effects of ketones on the developing heart. Poorly controlled Type 1 diabetes is a well-known cause of elevated ketones that is linked to adverse pregnancy outcomes including congenital anomalies [15]. We contend that it is important to consider whether these consequences are related to alterations in the overall diabetic milieu or to ketones alone. High ketones during pregnancy usually represent more extreme undernutrition or diabetic states, which are associated with exaggerated perturbations in many fuels, not just ketones. It is also important to consider the timing of the ketone exposure in the context of offspring development. We have previously shown that exposure to hyperglycemia during early embryogenesis is associated with teratogenesis, even in the absence of maternal diabetes or ketosis [50]. Others have demonstrated that alterations in morphogenesis can be produced by excess exposure to B-hydroxybutyrate as well [15,51]. Our experimental model is different in that it mimics gestational diabetes in humans, which occurs in the second half of pregnancy after morphogenesis is complete. Additionally, insulin was administered to keep glucose levels in a target range of 200–400 mg/dl and ketosis to a minimum. Over the course of ten years, we have not identified an increase in congenital heart defects; rather, we consistently find cardiometabolic dysfunction in the hearts of offspring exposed to late gestation maternal diabetes and HFD [10,12,13,23]. This correlates with differences in pregestational and late-gestation exposures in humans.

While this study confirms that insulin-resistant neonatal myocardium can utilize ketones as an alternative source, whether ketones are useful to help reverse the permanent effects of glucolipotoxicity during this critical window of development should be further examined. Interestingly, clinical studies revealed that the supplementation of ketones is beneficial for patients with chronic heart failure [52]. Indeed, ATP production following ketone administration was found to nearly triple in patients with heart failure [53]. Pharmacological interventions that target metabolic syndrome indirectly promote ketogenesis, which also appears to reduce the risk of adverse cardiovascular events [54,55]. Although the clinical trials with ketones in adult patients seem promising, similar studies are required to examine whether supplementing ketones to high-risk infants would be safe and beneficial for neonatal cardiomyopathy.

## 4. Materials and Methods

### 4.1. Animal Care

This study followed the guidelines set forth by the Animal Welfare Act and the National Institutes of Health *Guide for the Care and Use of Laboratory Animals* and was under approval from the Sanford Research Institutional Animal Care and Use Committee (Protocol #170-06-23B). All animals were housed in a temperature-controlled, light–dark cycled facility with free access to water and chow. Female Sprague–Dawley rats (Envigo, Indianapolis, IN) received control (TD2018 Teklad, Envigo; 18% fat, 24% protein, 58% carbohydrates) or HFD (TD95217 custom diet Teklad, Envigo; 40% fat, 19% protein, 41% carbohydrates) for at least 28 days prior to breeding to simulate a dietary “lifestyle.” Diets were selected to equate commonly attainable low-fat diets (18% of calories as fat) or HFD (40% of calories as fat) with more saturated and monounsaturated fat content. Omega 6:3 ratios were similar between diets. Female rats were bred with healthy male rats and fed a control diet and monitored by daily vaginal swab for spermatozoa. When spermatozoa were first present, timed pregnancy started as embryonic day 0 (E0). After confirming the pregnancy with an ultrasound, dams received an intraperitoneal injection of either citrate-buffered saline (Thomas Scientific, Swedesboro, NJ) diluent or 65 mg/kg streptozotocin (Sigma-Aldrich, Inc., St. Louis, MO, USA) to induce late gestation diabetes on E14. With a goal to keep blood glucose levels at 200–400 mg/dL, dams were partially treated with sliding scale insulin (regular and glargine, Eli Lilly and Co., Indianapolis, IN) two times per day. Whole blood sampling from a tail nick was done to measure glucose at least twice daily and ketones (βHOB) daily (Precision Xtra glucometer and ketone meter, Abbott Laboratories, Abbott Park, IL, USA). Dams with blood glucose < 200 mg/dL within 48 h after streptozotocin were excluded from the study. Although this model induces maternal diabetes by streptozotocin-mediated pancreatic damage, we have consistently shown that the developing offspring, our experimental subjects, are exposed to maternal hyperglycemia, hyperlipidemia, and fetal hyperinsulinemia in the last 1/3 of pregnancy [9,11,12,13,56]. Dams were allowed to deliver spontaneously in order to yield offspring of both sexes from two distinct groups: controls and combination exposed (diabetes + HFD). On postnatal day 1 (P1), offspring hearts (*n* = 10–12 litter per group and each group comprised 5–6 male and female hearts) were collected under 5% isoflurane anesthesia and immediately used for the isolation of NRCM or snap frozen in liquid nitrogen and stored at −80 °C until analysis. Maternal and offspring characteristics are given in Supplementary Tables S1 and S2. 

### 4.2. Isolation of Neonatal Rat Cardiomyocytes (NRCM)

Isolation of NRCM was done as previously detailed [9,10,13]. Briefly, hearts collected on P1 were transferred to Hank’s Balanced Salt Solution on ice. After removing atria, ventricles were minced and digested with 0.1% trypsin with 0.02% DNase I (in 0.15 M NaCl) via 5–6 alternating cycles of stirring (5 min at 50 rpm) followed by trituration at 1–2 mL/s for 5 min. Trypsin/DNase I mix was deactivated with bovine serum (BS) before centrifuging cells at 1600 rpm at 22 °C for 10 min. Cell pellets were resuspended in DMEM-1 supplemented with 10% BS and 1% penicillin/streptomycin with 0.0002% DNase I. Cells were seeded to uncoated 35 mm dishes, and incubated for 1 h in humidified 37 °C, 5% CO_2_ to allow fibroblast attachment. NRCM were then gently detached, resuspended in DMEM-1, and counted with a hemocytometer using Trypan Blue before seeding to 0.1% gelatin-coated Seahorse XFe24 V7 PS cell culture microplates (Agilent, Santa Clara, CA) at 150,000 cells/well for extracellular flux analyses. NRCM were allowed to adhere overnight (12–16 h) before experiments.

### 4.3. Ketone Stress Test (KST) in Isolated NRCM

**Figure 10:**
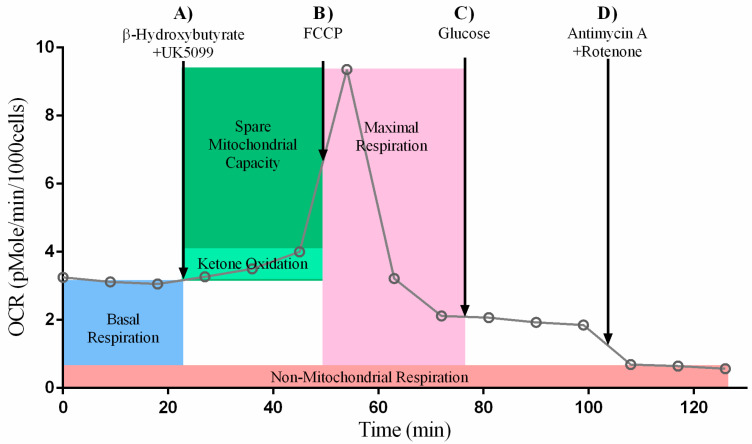
Schematic representation of the ketone stress test (KST) for assessing mitochondrial respiration in terms of oxygen consumption rate (OCR).

Isolated NRCM on gelatin-coated XFe24 microplates were washed with XF DMEM media (Agilent, Santa Clara, CA, USA) and placed in a 37 °C incubator without CO_2_ for 1 h to degas. Analyses were run on a Seahorse XFe24 analyzer (Agilent, Santa Clara, CA, USA) after validating seeding density and drug dosing according to the manufacturer’s recommendation, as previously detailed [9,10,13,14]. Temperature and pH of the media was adjusted to 37 °C and 7.4, respectively. Oxygen consumption rates (OCR) and extracellular acidification rates (ECAR) were measured at baseline and following injections with: (A) 1.5 µM UK5099, a pyruvate inhibitor (R&D Systems, Minneapolis, MN, USA) and 0, 1.5, or 4.5 mM βHOB (Sigma, St. Louis, MO, USA); (B) 0.3 µM carbonyl cyanide p-trifluoromethoxyphenyl-hydrazone (FCCP) (Sigma, St. Louis, MO, USA); (C) 10 mM D-(+)-Glucose (Sigma, St. Louis, MO, USA); (D) 2 µM Rotenone (Sigma, St. Louis, MO, USA), 4 µM Antimycin A (Sigma, St. Louis, MO, USA), and 2 µM Hoechst (AnaSpec, Fremont, CA, USA) to stain remaining live cells at the end of the run for normalization. A schematic representation of the protocol is shown in Figure 2 and Figure 10. After measurements, the cells were imaged and counted using Cytation 1 Cell Imaging Multi-Mode Reader (Agilent, Santa Clara, CA, USA) for data normalization to cell count. Proton efflux rates (PER) were calculated as initially described by Mookerjee et al. [57] and are shown in Supplementary Table S3.

### 4.4. Quantitative Real Time PCR 

RNA was extracted from newborn (P1) rat ventricles using the RNeasy Fibrous Tissue Mini kit (Qiagen, Germantown, MD, USA) following the manufacturer’s protocol. RNA integrity was assessed by electropherograms using 2100 BioAnalyzer (Agilent Technologies, Santa Clara, CA, USA) and demonstrated RNA Integrity Numbers of 9.2–10 (average = 9.8). RNA concentration was measured by Epoch spectrophotometer (BioTek, Winooski, VT, USA). Complementary DNA (cDNA) was synthesized using iScript cDNA Synthesis Kit and T100 Thermal Cycler (Bio-Rad, Hercules, CA, USA). Quantitative PCR (qPCR) was performed by TaqMan approach with Absolute Blue qPCR Mix using an ABI7500 qPCR system (ThermoFisher, Waltham, MA, USA). Beta-2-microglobulin (*B2m*) was used as the reference gene. *B2m*, *Hmgcs2*, *Pparg*, and *Cpt1a* probe/primer sets were obtained from ThermoFisher (Waltham, MA, USA), and *Pgc1a* probe/primer set was obtained from Integrated DNA Technologies (Coralville, IA, USA). Details are given in Supplementary Table S4.

### 4.5. Statistical Analysis

All statistical analyses were done with Prism 9 (GraphPad Software). Further information, including sample sizes and number of replicates, is provided in the legends accompanying each figure. Two-way ANOVA was used to detect the effects of in utero diabetes + HFD (combination) exposure, ketone dosing, and interactions. When a significant group or interaction effect was present, one-way ANOVA with Tukey post-test for individual group comparison was also determined. Unpaired *t*-test followed by a Mann–Whitney *U* test was used to examine group differences including maternal and neonatal characteristics, gene expression, and sex-specific comparisons. For all statistical tests, *p* < 0.05 was considered statistically significant.

## 5. Conclusions

This study used a novel KST to analyze real-time ketone body metabolism in NRCM. Importantly, we used this assay to show that even though offspring exposed to diabetic pregnancy and HFD have myocardial mitochondrial dysfunction, they can still metabolize ketones, which are known to be protective for the diabetic and failing heart in humans. Considering (i) the alarming rate of myocardial dysfunction in the infants exposed to maternal diabetes and obesity, as well as (ii) the role of ketone body metabolism in the failing hearts, this study provides critical information towards understanding the potential use of ketones for refractory cases of neonatal cardiomyopathy in these high-risk infants.

## Data Availability

Any data not included in the manuscript or supplementary file are available from the corresponding author upon request.

## Supplementary Materials

The following supporting information can be downloaded at: https://www.mdpi.com/article/10.3390/ijms24043684/s1.
