# Supplementary material for: Impact of Prenatal Exposure to Maternal Diabetes and High-Fat Diet on Postnatal Myocardial Ketone Body Metabolism in Rats
Source: Int J Mol Sci. 2023 Feb 12;24(4):3684. doi: 10.3390/ijms24043684 (PMC9967912; doi:10.3390/ijms24043684)
Supplement: Supplementary file 1 [file ijms-24-03684-s001.zip › ijms-2195660-supplementary.pdf]

Supplementary Tables and Figure

Table S1. Model's maternal characteristics.

**Parameters**	**Controls (mean±SD)**	**Combination exposed (mean±SD)**	**P value**
Baseline weight (g)	208.7±30.34	190.6±29.52	0.105
Post-Diet weight (g)	229.7±29.99	274.2±12.45*	0.0017
Blood glucose (mg/dL)	89.67±10.06	319.9±57.77*	<0.0001
Blood ketones (mM/L)	0.541±0.041	0.681±0.177*	0.017
Litter size (pups)	13.56±2.091	12.73±2.257	0.279

* values are significantly different from control group and the significance accepted at $P<0.05$ (n=10-13) in each group. SD-standard deviation.

Table S2: Model's offspring characteristics

**Parameter**	**Controls**	**Combination exposed**	**P value**
Body weight (g)	6.15±0.761	5.81±0.672*	0.008
Heart weight (g)	0.043±0.007	0.043±0.006	0.723
Heart:body weight ratio	0.0071±0.0011	0.0075±0.0009*	0.044

* values are significantly different from control group and the significance accepted at $P<0.05$ (n=10-13) in each group. SD-standard deviation.

Table S3

Calculations of proton efflux rate (PER) and PER related variables [25]

**Sl. No**	**Parameter**	**Equation**
1	PER (pmol H+/min)	ECAR (mpH/min) x BF (mmol/L/pH) x Geometric volume (µl) x Kvol
2	PER_{Gluc}	PER - PER_{mito}
3	PER_{mito} or PER_{CO2}	CCF x OCR_{mito} (whereas CCF = CO2 contribution factor which is 0.6; $OCR_{mito} = OCR_{basal} – OCR_{Rote/AA}$)
4	$PER_{Lactate}$	PER_{Gluc}-PER_{mito}
5	Baseline fraction of $PER_{Lactate}$	$PER_{Lactate}/PER_{Glucose}$

PER related variables

Sl. No	Variable	Explanation
1	Extracellular acidification rate (ECAR) (mPH/min)	Rate data measured during an assay, reported as the rate of change in mpH in an assay well.
2	Buffer factor (BF) (mmol/L/pH)	Measure of the *in situ* buffer capacity obtained in XF instruments, and accounts for buffer capacity of both medium and sensor system. Calculated BF is 2.689
3	Geometric volume (μl)	Physical volume of the measurement microchamber assuming its completely sealed. Calculated geometric volume is 5.65 μl
4	Volume scaling factor (Kvol)	Empirically derived scaling factor used to account for total proton production in the measurement chamber. Calculated Kvol is 1.19

PER- proton efflux rate; ECAR-extracellular acidification rate; OCR-oxygen consumption rate.

Table S4: List of Primers used for qPCR

Gene	Manufacturer	Reference Sequence	Assay ID	Amplicon sequence
B2m	Thermo Fisher	NM_012512.2	Rn00560865_m1	Amplicon: CTGCTGACCGGACCGGCACGATGGCTCGCTCGGTGACCGTGATCT TTCTGGTGCTTGTCTCTCTGGCCGTCGTGCTTGCCATTCAGAAAACT CCCCAAATTCAAGTGTACTCTCGCCATCCACCGGAGAATGGGAAGCCCAACTTCCTCA ACTGCTACGTGTCTCAGTTCCACCCACCTCAAATAGAAATTGAGCTACTGAAGAATGG AAAGAAGATACCAAATATCGAG ATGTCA
Hmgcs2	Thermo Fisher	NM_173094.2	Rn00597339_m1	Amplicon: ACATGTACAC CTCGTCCCTC TACGGGTGCC TGGCCTCACT TCTCTCCCAC CACTCTGCCCAAGAATTGGCCGGCTCCAGGATTGGAGCAT TCTCCTACGG CTCAG
Pparγ	Thermo Fisher	NM_001145366.1	Rn00440945_m1	Amplicon: TGACTTTATG GAGCCTAAGT TTGAGTTTGC TGTGAAGTTC AATGCACTGG AATTAGATGA CAGTGACTTG GCCATATTTA TAGCTGTCAT TATTCTCAGT GGAGACCGCC CAGGCTTGCT GAACGTGAAG CCCATCGAGG ACATCCAAGA CAACCTGCTG CAGGCCCTGG AACTCCAGCT GAAGCTGAAC CACCCGGAGT CCTCCCAGCTG
Cpt1α	Thermo Fisher	NM_031559.2	Rn00580702_m1	Amplicon: TAAAGGAGAC ACCAACCCCA ACATCCCTAA GCCCACAAGG CTACAATGGG ACATTCCAGG AGAGTGCCAG GAGGTCATAG ATGCATCCCT GAGCAGCGCC AGTCTTTTGG CAAATGATGT GGACCTGCA
Pgc1α	Integrated DNA Technologies	NM_031347	Rn.PT.58.37655048	Probe: 5′/56-FAM/CATACACAA/ZEN/CCGCAG TCGCAACATG/3IABkGQ/-3′<br>P1: 5′-GGTCAGAGGAAGAGATAAAGTTGT-3′<br>P2: 5′-ACCCACAGAGAACAGAAACAG-3′

Table S5: Effect of UK5099 on neonatal cardiomyocyte respiration

Group	Mean OCR (pMol/min/1,000 cells)	SEM	Different from basal (p value)	Different from UK099 alone (p value)
Control Basal	4.291	0.398	NA	P>0.9999
Control UK5099	4.261	0.505	NS, P>0.9999	NA
Control +1.5mM ßOHB	4.657	0.550	NS, P=0.9976	NS, P=0.9960
Control + 4.5mM ßOHB	4.423	0.394	NS, P>0.9999	NS, P>0.9999
Combination Basal	3.943	0.170	NS, P=0.9985	NS, P=0.9991
Combination UK5099	3.668	0.319	NS, P=0.9952	NS, P=0.9632
Combination + 1.5mM ßOHB	4.083	0.336	NS, P>0.9999	NS, P>0.9999
Combination + 4.5mM ßOHB	4.362	0.281	NS, P>0.9999	NS, P>0.9999

UK5099- mitochondrial pyruvate carrier inhibitor; OCR- oxygen consumption rate; SEM- standard error of the mean; ßOHB-beta-hydroxybutyrate; NA- not applicable; NS- not significant from untreated control by 1-way ANOVA.

Table S6: Effect of UK5099 on neonatal cardiomyocyte glycolysis

Group	Mean ECAR (pMol/min/1,000 cells)	SEM	Different from basal (p value)	Different from UK099 alone (p value)
Control Basal	0.1837	0.0215	NA	NS, P>0.9999
Control UK5099	0.1909	0.0260	NS, P>0.9999	NA
Control +1.5mM ßOHB	0.1755	0.0253	NS, P>0.9999	NS, P>0.9999
Control + 4.5mM ßOHB	0.1575	0.0149	NS, P=0.9997	NS, P=9986
Combination Basal	0.2706	0.0544	NS, P=0.7724	NS, P=0.8409
Combination UK5099	0.2609	0.0397	NS, P=0.8614	NS, P=0.9124
Combination + 1.5mM ßOHB	0.2374	0.0501	NS, P=0.9789	NS, P=0.9905
Combination + 4.5mM ßOHB	0.2594	0.0653	NS, P=0.8730	NS, P=0.9211

UK5099-mitochondrial pyruvate carrier inhibitor; ECAR- extracellular acidification rate; SEM- standard error of the mean; ßOHB-beta-hydroxybutyrate; NA- not applicable; NS- not significant by one-way ANOVA.

Table S7: Control and combination exposure differences in KST by sex

Parameter	Sex	0 mM Controls	0 mM Combination	1.5 mM Controls	1.5 mM Combination	4.5 mM Controls	4.5 mM Combination	P value (0 mM)	P value (1.5 mM)	P value (4.5 mM)
Basal respiration	M	3.619±0.433	3.177±0.201	3.414±0.504	2.989±0.247	3.282±0.502	2.935±0.260	0.428	0.662	0.930
	F	3.198±0.425	3.124±0.274	3.097±0.424	2.904±0.264	3.159±0.417	3.106±0.361	>0.99	>0.99	0.841
Ketone oxidation	M	-0.091±0.337	-0.063±0.297	-0.409±0.361	-0.270±0.47	0.205±0.189	0.656±0.380	0.792	0.662	0.792
	F	-0.091±0.099	-0.585±0.397	0.633±0.512	0.694±0.269	0.288±0.117	0.365±0.482	0.547	0.841	0.420
Maximal respiration	M	6.377±0.849	7.496±1.117	6.614±0.707	5.992±0.963	8.591±1.095	8.237±0.898	0.662	0.662	0.662
	F	7.795±1.481	5.267±1.390	7.73±1.297	6.010±0.997	8.762±1.710	8.372±0.861	0.150	0.420	>0.99
Spare respiratory capacity	M	2.755±0.952	4.32±1.114	3.197±0.662	3.003±1.064	5.306±1.062	5.302±0.787	0.329	0.930	0.930
	F	4.539±1.137	2.143±1.484	4.576±1.117	3.106±1.114	5.547±1.392	5.265±0.751	0.222	0.690	>0.99
Glucose mediated OCR	M	1.030±0.487	1.152±0.309	1.362±0.439	1.434±0.5276	1.578±0.469	2.412±0.868 [a]	>0.99	>0.99	0.737
	F	1.098±0.357	0.964±0.607	0.9860±1.280	1.766±1.271	1.786±0.410	2.098±0.807	>0.99	0.847	>0.99
Non-mitochon drial respiration	M	0.957±0.214	0.824±0.117	1.015±0.158	1.012±0.126	1.148±0.214	1.066±0.105	0.329	0.930	0.536
	F	0.927±0.159	0.760±0.163	1.028±0.178	0.980±0.130	0.968±0.171	0.778±0.139	0.690	>0.99	0.222
Basal ECAR	M	0.181±0.07	0.25±0.158					0.836		
	F	0.186±0.08	0.29±0.198					0.624		
Ketone mediated ECAR	M	0.003±0.02	0.012±0.04	-0.011±0.02	-0.04±0.06	-0.02±0.019	-0.010±0.04	0.99	0.77	0.99
	F	0.012±0.025	-0.036±0.058	0.001±0.030	-0.023±0.039	-0.03±0.048	-0.011±0.03	0.46	0.928	0.925
Maximal	M	0.050±0.029	0.0094±0.029	0.061±0.018	-0.008±0.025	0.057±0.018	0.140±0.078	0.329	0.125	0.930

ECAR	F	-0.014±0.013	-0.038±0.029	-0.012±0.020	0.0121±0.038	-0.0118±0.02	0.111±0.058	0.309	0.690	0.095
Glucose mediated ECAR	M	0.11±0.09	0.091±0.06	0.0125±0.058	0.051±0.034	0.131±0.077	0.241±0.251 [b]	>0.99	0.99	0.913
	F	0.05±0.04	-0.0008±0.05	0.073±0.04	0.124±0.14	0.07±0.09	0.221±0.193	0.99	>0.99	0.72
Anaerobic ECAR	M	0.592±0.142	0.865±0.181	0.4905±0.102	0.651±0.162	0.696±0.164	0.826±0.171	0.125	0.428	0.662
	F	0.874±0.215	0.688±0.147	0.660±0.161	0.457±0.125	0.772±0.215	0.827±0.145 [*]	0.690	0.547	0.841
Basal PER	M	1.655±0.260	1.674±0.252	NA	NA	NA	NA	0.329	NA	NA
	F	1.698±0.331	1.924±0.519	NA	NA	NA	NA	0.690	NA	NA
B-HOB PER	M	1.676±0.300	1.768±0.148	1.512±0.282	1.442±0.085	1.499±0.223	1.468±0.162	0.329	0.662	0.792
	F	1.819±0.412	1.872±0.186	1.707±0.410	1.723±0.233	1.359±0.153	1.799±0.275	0.547	0.547	0.222
FCCP PER	M	7.054±1.231	8.947±2.063	6.127±0.999	5.411±0.730	8.002±1.511	7.914±1.605	0.536	0.609	>0.99
	F	9.672±2.254	7.676±1.672	7.723±1.741	5.163±0.907	8.737±2.125	8.311±1.516	0.547	0.690	>0.99
Glucose PER	M	2.691±0.459	2.32±0.327	2.846±0.423	2.260±0.264	2.854±0.419	3.219±0.668	>0.99	0.536	0.792
	F	2.223±0.277	2.081±0.147	2.366±0.267	2.442±0.495	2.442±0.313	3.256±0.465	>0.99	0.841	0.222
Rot/AA PER	M	2.11±0.374	1.828±0.301	2.211±0.323	1.681±0.189	2.176±0.310	2.523±0.576	0.792	0.428	0.792
	F	1.566±0.256	1.70±0.118	1.582±0.223	1.877±0.374	1.591±0.159	2.577±0.371	0.690	0.841	0.095
Glucose mediated $PER_{Lactate}$	M	1.984±0.408	1.629±0.262	2.029±0.358	1.441±0.144	1.922±0.356	1.886±0.502	0.930	0.329	0.792
	F	1.574±0.192	1.502±0.109	1.446±0.200	1.384±0.235	1.35±0.258	1.997±0.354	0.841	0.841	0.222
Glucose mediated PER_{CO2}	M	0.706±0.065	0.690±0.08	0.817±0.107	0.818±0.161	0.946±0.115	1.33±0.184	>0.99	0.662	0.246
	F	0.658±0.096	0.578±0.163	0.920±0.115	0.731±0.121	1.072±0.109	1.259±0.216	0.420	0.285	0.690
Maximal $PER_{Lactate}$	M	3.310±1.877	5.363±3.348	2.318±1.449	3.337±3.052	3.173±3.735	3.294±3.171	0.983	>0.99	>0.99
	F	5.011±3.088	4.406±2.695	3.145±2.438	1.630±1.235	3.477±3.216	2.731±2.687	>0.99	0.99	>0.99

Maximal PER_{CO2}	M	3.735±1.251	3.596±1.733	3.803±1.353	3.934±1.303	4.822±2.062	4.604±1.140	>0.99	>0.99	>0.99
	F	4.652±1.998	3.241±1.808	4.646±1.743	3.258±1.214	5.254±2.293	5.354±1.071	0.963	0.967	>0.99
Baseline fraction PER lactate	M	0.713±0.034	0.697±0.024	0.704±0.031	0.643±0.041	0.660±0.044	0.562±0.036	0.536	0.329	0.125
	F	0.7049±0.019	0.733±0.058	0.607±0.037	0.591±0.050	0.544±0.039	0.606±0.044	0.309	>0.99	0.420

Values are represented as mean±SD (n=6 for control males and n=5 for combination males; n=5 for control females and combination females). * indicates values are significantly different from 0 mM combination females (p=0.044). [a] indicates values shows a trend towards significance (p=0.088) compared to 0mM control males. [b] indicates values are significantly different from 0 mM combination males (p=0.049). OCR-oxygen consumption rate; ECAR-extracellular acidification rate; PER-proton efflux rate; Rot-Rotenone; AA-Antimycin A; βHOB- ß-hydroxybutyrate; FCCP- Carbonyl cyanide 4-(trifluoromethoxy)phenylhydrazone, M-male; F-female. mM refers to the progressive amount of βHOB supplied.

Table S8: Sex-specific differences in KST

Parameter	P value (male vs female; 0mM Controls)	P value male vs female (0mM Combination)	P value (male vs female; 1.5 mM Controls)	P value (male vs female; 1.5 mM Combination)	P value (male vs female; 4.5 mM Controls)	P value (male vs female 4.5 mM; Combination)
Basal respiration	0.428	0.841	0.662	0.841	>0.999	0.690
Ketone oxidation	>0.999	0.3095	0.329	0.150	>0.999	0.420
Maximal respiration	0.662	0.309	0.536	0.841	0.930	0.841
Spare respiratory capacity	0.246	0.309	0.329	>0.999	0.930	0.841
Glucose mediated OCR	>0.999	>0.999	0.992	0.998	>0.999	>0.999
Non-mitochondrial respiration	0.930	0.547	0.792	0.841	0.536	0.222
Basal ECAR	>0.999	0.965				
Ketone mediated ECAR	>0.999	0.744	>0.999	0.849	>0.999	>0.999
Maximal ECAR	0.246	0.690	0.246	0.547	>0.999	>0.999
Glucose mediated ECAR	0.999	0.980	0.999	0.997	0.999	>0.999
Anaerobic ECAR	0.082 (♂< ♀)	0.222	*0.030* (♂<♀)	0.841	0.051 (♂<♀)	>0.999
Basal PER	0.792	0.309				
B-OHB PER	0.930	0.690	0.930	0.690	>0.999	0.420
FCCP PER	0.428	0.841	0.536	0.904	0.930	>0.999
Glucose PER	0.536	>0.999	0.428	0.841	0.662	0.841
Rot/AA PER	0.428	0.246	0.246	0.841	0.841	0.690
Glucose mediated PER Lactate	0.662	>0.999	0.246	0.841	0.246	0.690
Glucose mediated PER CO2	0.662	0.309	0.662	0.730	0.428	>0.999

Maximal PER Lactate	>0.999	>0.999	>0.999	>0.999	0.997	>0.999
Maximal PER CO2	0.998	>0.999	0.998	>0.999	>0.999	0.998
Baseline fraction PER lactate	0.792	0.420	0.082 (♂>♀)	0.690	0.051 (♂>♀)	0.420

OCR-oxygen consumption rate; ECAR-extracellular acidification rate; PER-proton efflux rate; Rot-Rotenone; AA-Antimycin A; βHOB- ß-hydroxybutyrate; FCCP- Carbonyl cyanide 4-(trifluoromethoxy)phenylhydrazone.

Table S9: Sex-specific differences in gene expression

Parameter	Sex	Fold change		P value (male vs female; controls)	P value (male vs female; combination)
		Control	Combination		
Cpt1a (Fold change)	M	0.258±1.445	-0.341±1.169	0.937	0.142
	F	1.7±0.642	0.1867±1.56		
Hmgcs2	M	-0.360±1.27	1.84±0.688	0.937	0.002 (♂>♀)
	F	0.248±1.476	-2.442±1.195		
Bdh1	M	-0.515±1.771	1.812±0.370	>0.999	0.002 (♂>♀)
	F	-0.515±1.702	-0.566±1.455		
Pparg	M	-0.066±1.479	1.695±0.540	0.974	0.225
	F	-0.338±1.139	0.690±1.396		
Pgc1a	M	-0.036±1.372	2.238±0.459	0.818	0.240
	F	-0.798±1.437	4.122±2.430		

Cpt1a- carnitine palmitoyl transferase 1a; *Hmgcs2*-3-hydroxy-3-methylglutaryl-CoA synthase 2**;** *Bdh1*- β-hydroxy butyrate dehydrogenase; *Pparg*-Peroxisome proliferator-activated receptor γ; Pgc*1a*-Peroxisome proliferator-activated receptor γ coactivator-1.

Figure S1. Non-mitochondrial respiration and Basal PER.tif

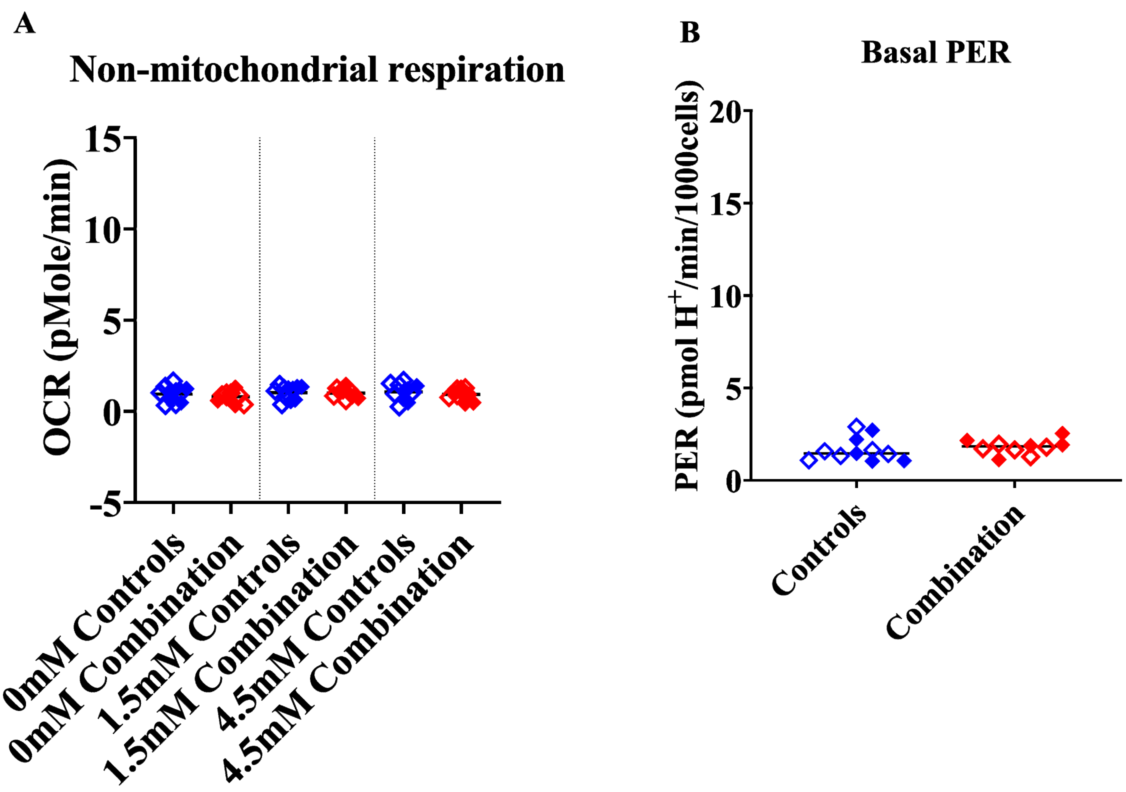

Figure S1. Non-mitochondrial respiration and Basal PER were not different in control and combination exposed groups. (A) Non-mitochondrial respiration, (B) Basal PER. Open symbols in the graph indicate males and filled symbols indicate females. Bars represent mean OCR (n=10-11 litter per group).
